# Heat of Formation of Titanium Tetraiodide

**DOI:** 10.6028/jres.063A.009

**Published:** 1959-10-01

**Authors:** W. H. Johnson, A. A. Gilliland, E. J. Prosen

## Abstract

The heat of formation of titanium tetraiodide was determined relative to that of titanium tetrabromide by comparison of their heats of hydrolysis in dilute sulfuric acid. The difference in the heats of formation may be expressed by the equation:

$$\begin{array}{c} {\text{TiI}}_{4}\left(c\right)+2{\text{Br}}_{2}\left(\text{liq}\right)=\text{TiBr}\left(c\right)+2I_{2}\left(c\right),\\ \Delta H\left(25{}^{\circ}C\right)=-230.91\pm 0.75\;\text{kj}/\text{mole}\left(-55.19\pm 0.18\;\text{kcal}/\text{mole}\right). \end{array}$$

By taking the heat of formation of TiBr_4_(c) as −616.72 ±4.60 kj/mole, the heat of formation of TiI_4_(c) is calculated to be −385.81 ±4.64 kj/mole (−91.21 ±1.11 kcal/mole). The heats of hydrolysis of TiBr_4_ and TiCl_4_ were similarly measured; the value obtained for the difference (186.77 ±1.34 kj/mole) is in good agreement with the difference between the directly determined heats of formation (187.11 ±5.35 kj/mole).

## 1. Introduction

There are no experimental values available in the literature for the heat of formation of titanium tetraiodide. Brewer, Bromley, Gilles, and Lofgren [1]^1^ calculated the heat of formation from estimated heats of solution and lattice-energy calculations. They obtained −101 ±10 kcal/mole for the heat of formation of TiI_4_ at 25° C.

The direct combination of elemental titanium with gaseous iodine is not feasible because of the low vapor pressure of iodine at ordinary temperatures; at higher temperatures, the tetraiodide tends to decompose into TiI_2_ and TiI_3_ [2].

The heats of formation of titanium tetrachloride and titanium tetrabromide have recently been determined by the direct combination of the elements in a calorimeter [3, 4].

It is possible to hydrolyze TiI_4_, as well as TiCl_4_ and TiBr_4_, in dilute sulfuric acid. We can assume that the final state of the Ti^4+^ ion is the same in each case and that the differences in the heats of hydrolysis are measures of the differences in the heats of formation. We may, therefore, compare the heats of hydrolysis of TiI_4_ and TiBr_4_ and obtain the heat of formation of TiI_4_ relative to that of TiBr_4_. We may also compare the heats of hydrolysis of TiBr_4_ and TiCl_4_ and obtain the difference between their heats of formation. This value should agree with the difference between the directly determined heats of formation.

The present investigation is comprised of two parts. The first part consists of the measurement of the heats of hydrolysis of TiI_4_ and TiBr_4_ and the calculation of the heat of formation of TiI_4_ relative to that of TiBr_4_. The second part includes the measurement of the heats of hydrolysis of TiBr_4_ and TiCl_4_ and the comparison of the difference between these heats of hydrolysis with the difference between the heats of formation.

## 2. Source and Purity of Samples

The TiCl_4_, TiBr_4_, and TiI_4_ were prepared by the Inorganic Chemistry Section of the Chemistry Division. The purity of the TiCl_4_ and TiBr_4_ was determined to be 99.999 and 99.998 mole percent, respectively, from calorimetric freezing-point measurements conducted by George T. Furukawa of the Thermodynamics Section of the Heat Division. The purity of the TiI_4_ was determined by analysis to be 99.91 mole percent; this analysis was performed by the Inorganic Chemistry Section of the Chemistry Division.

The 1–*N* sulfuric acid solution used as the calorimetric fluid for the hydrolysis experiments was prepared from concentrated, reagent-grade acid.

The hydrobromic and hydriodic acids were redistilled from reagent-grade solutions; the constantboiling fractions were collected under nitrogen and stored in darkness in completely filled, glass-stoppered bottles. The hydrochloric acid was taken directly from a fresh bottle of reagent-grade acid.

The TiCl_4_ and TiBr_4_ were distilled in vacuum into Pyrex glass bulbs, and the bulbs sealed. Soft glass bulbs were filled with the solid TiI_4_ in a helium atmosphere. The bulbs were then fitted with polyethylene caps, removed from the helium atmosphere, cooled in ice, and sealed. The hydrochloric, hydrobromic, and hydriodic acid solutions were placed in soft glass bulbs by means of a funnel drawn down to a capillary. They were then cooled in ice and sealed.

The quantity of each of the titanium tetrahalide samples was determined by weighing the bulbs before and after filling. The quantities of the hydrobromic and hydriodic acid samples were determined from the mass of the sample and the concentration of the stock solution. The quantities of the hydrochloric acid samples were determined by analysis of the resulting calorimetric solution.

## 3. Apparatus and Procedure

**Figure 1:**
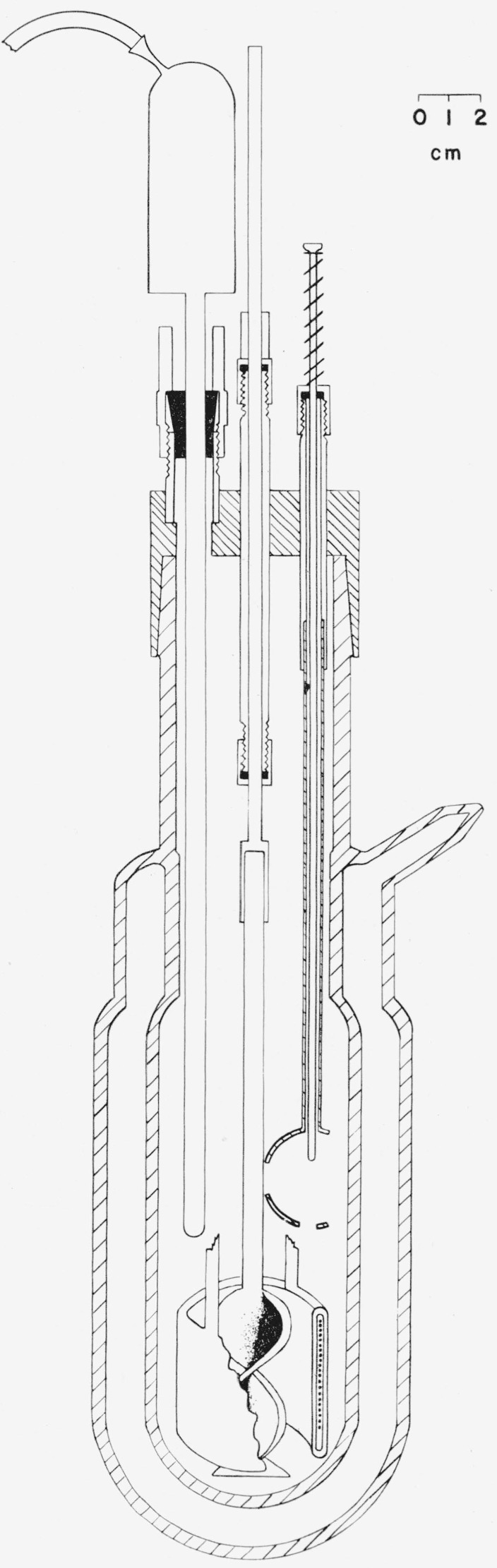
Glass calorimeter.

The calorimeter used for measurement of the heats of hydrolysis and dilution is shown in figure 1. It consists of a glass vessel having a silvered, evacuated jacket, a glass-enclosed manganin heating coil, a glass stirrer, a glass bulb-crusher, and a glass-enclosed platinum resistance thermometer. The upper part of the vessel consists of a 60/35 standard-taper joint; the volume of the enlarged portion is approximately 500 ml.

The heating coil, stirrer, bulb-crusher, and platinum thermometer are all supported by the brass head. The resistance of the heating coil is 124.33 ohms. The heater leads, which pass through the supporting tubes, have branch points, similar to a 4-lead thermohm, located approximately midway between the calorimeter and jacket boundaries. The stirrer shaft is constructed of stainless steel and passes through a stainless steel tube fitted at both ends with Teflon gaskets which serve as bearings. The glass stirrer is sealed into a stainless steel cup welded to the stirrer shaft. The calorimetric assembly was immersed in a thermostatically-controlled water bath during each experiment.

The apparatus for measurement of calorimeter temperatures, for calibration with electrical energy, and the details of calorimetric procedures have been described in previous papers [3, 4].

In order to compare the heats of hydrolysis of TiBr_4_ and TiI_4_, it was necessary that the composition of the end solution be essentially the same in each case. For this reason 0.04 mole of hydriodic acid was added to the sulfuric acid solution for the TiBr_4_ hydrolysis experiments, and 0.04 mole of hydrobromic acid was added to the solution for the TiI_4_ hydrolysis experiments. A similar procedure was used in the determination of the heats of hydrolysis of TiBr_4_ and TiCl_4_.

The solutions resulting from the TiI_4_ hydrolysis experiments were carefully removed from the calorimeter and neutralized with ammonium hydroxide solution. The solution was boiled to coagulate the precipitate which was then filtered and ignited to constant weight and weighed as TiO_2_. The average ratio of the TiO_2_ found to the theoretical quantity based upon the weight of sample was 1.0022 ± 0.0028.

The solutions resulting from the TiI_4_ hydrolysis experiments and from the hydriodic acid dilution experiments varied in color from faintly amber to brown. After standing for a few hours they became noticeably darker in color, presumably because of oxidation of the hydriodic acid.

The solutions resulting from the TiBr_4_ hydrolysis experiments and the hydrobromic acid dulition experiments were clear, but usually turned very faintly amber after standing, probably because of oxidation of the hydriodic and hydrobromic acids.

The solutions resulting from the TiCl_4_ hydrolysis experiments were very slightly turbid, but they became clear after standing for a few hours. This was probably caused by the rapidity of the reaction which, for some unknown reason, varied considerably among experiments. In some cases the violence of the reaction caused liquid to be splashed on the calorimeter walls above the level of the solutions, which made it necessary to reject the experiment.

## 4. Experimental Results

### 4.1. Hydrolysis of TiI_4_

The results of the electrical calibration experiments are given in table 1, where *E* is the electrical energy, in joules, added to the system; Δ*Rc* is the corrected temperature rise in ohms; and *E_s_* the energy equivalent of the “standard” calorimetric system in joules per ohm.

The results of the TiI_4_ hydrolysis experiments are given in table 2. The quantity Δ*e* is the deviation in joules per ohm of the electrical energy equivalent of the actual calorimetric system from that of the calibrated system. This deviation includes the heat capacity of the sample and of the glass bulb, less that of the empty bulb used in the calibration. The energy in joules, *g*, evolved by the process is obtained as the product of Δ*Rc* and the actual energy equivalent of the system. The heat of hydrolysis, *−*Δ*H* (27° C), is therefore the ratio of *q* to the number of moles of sample. For these calculations the heat capacities of TiI_4_(c) and Pyrex glass were taken as 0.235 and 0.795 j/g°C, respectively. For conversion to the conventional thermochemical calorie, one calorie has been taken as equivalent to 4.1840 j.

The heat of hydrolysis obtained in table 2 corresponds to the process:

$$\begin{array}{l} {\text{TiI}}_{4}\left(c\right)+\left[24H_{2}{\text{SO}}_{4}+4\text{HBr}+2600\;H_{2}O\right]\left(\text{soln}\right)=\\ \left[{\text{Ti}}^{4+}+4I^{-}+4H^{+}+4{\text{Br}}^{-}+24H_{2}{\text{SO}}_{4}+2600H_{2}O\right]\left(\text{soln}\right)\\ \Delta H\left(27{}^{\circ}C\right)=-217.81\pm 0.42\;\text{kj}/\text{mole}. \end{array}$$ (1)

### 4.2. Hydrolysis of TiBr_4_ in Sulfuric Acid Solution Containing Hydriodic Acid

The results of the electrical calibration and hydrolysis experiments are given in tables 3 and 4, respectively. The heat capacity of crystalline TiBr_4_ was taken as 0.358 j/g°C [5]. The resulting heat of hydrolysis corresponds to the process:

$$\begin{array}{l} {\text{TiBr}}_{4}\left(c\right)+\left[24H_{2}{\text{SO}}_{4}+4\text{HI}+2600\;H_{2}O\right]\left(\text{soln}\right)\\ \;=\left[{\text{Ti}}^{4+}+4I^{-}+4H^{+}+4{\text{Br}}^{-}+24\;H_{2}{\text{SO}}_{4}+2600\;H_{2}O\right]\left(\text{soln}\right),\\ \;\Delta H\left(27{}^{\circ}C\right)=-244.63\pm 0.20\;\text{kj}/\text{mole}. \end{array}$$ (2)

### 4.3. Heats of Dilution of Hydriodic and Hydro bromic Acids

The heats of dilution of the constant-boiling HI and HBr solutions in 1–*N* sulfuric acid are given in tables 5 and 6. Because of the small amount of energy evolved in these experiments, the actual calorimetric system was calibrated just prior to each experiment. The quantity *E_a_* denotes the energy equivalent of the actual calorimetric system. The heats of dilution correspond to the following reactions:

$$\begin{array}{c} \left[4\text{HI}+29.1\;H_{2}O\right]\left(\text{soln}\right)+\left[24H_{2}{\text{SO}}_{4}+2600\;H_{2}O\right]\left(\text{soln}\right)\\ =\left[4\text{HI}+24H_{2}{\text{SO}}_{4}+2629.1\;H_{2}O\right]\left(\text{soln}\right),\\ \Delta H\left(27{}^{\circ}C\right)=-11.256\pm 0.088\;\text{kj}, \end{array}$$ (3)

$$\begin{array}{c} \left[4\text{HBr}+20.1\;H_{2}O\right]\left(\text{soln}\right)+\left[24H_{2}{\text{SO}}_{4}+2600\;H_{2}O\right]\left(\text{soln}\right)\\ =\left[4\text{HBr}+24H_{2}{\text{SO}}_{4}+2620.1\;H_{2}O\right]\left(\text{soln}\right),\\ \Delta H\left(27{}^{\circ}C\right)=-31.97\pm 0.24\;\text{kj},\\ \Delta H\left(25{}^{\circ}C\right)=-31.54\pm 0.25\;\text{kj}. \end{array}$$ (4)

### 4.4. Heat Capacity of Hydrobromic and Hydriodic Acid Solutions

Because of the lack of data on the heat capacities of aqueous solutions of hydrobromic and hydriodic acid, these values were determined in separate experiments. The effective heat capacity of the empty calorimeter was determined by measuring the heat capacity when filled with pure water, and subtracting the known heat capacity of the water from the observed energy equivalent. The calorimeter was then filled with the same volume of the hydrobromic acid solution, weighed, and the energy equivalent determined. By subtracting the heat capacity of the calorimeter from the observed energy equivalent, the heat capacity of the hydrobromic acid solution was determined. The same procedure was used for determination of the heat capacity of the hydriodic acid solution.

The mean heat capacities obtained for aqueous hydrobromic and hydriodic acid in the range from 25° to 27° C were found to be:

$$\begin{array}{l} \left[\text{HBr}+5.0\;H_{2}O\right],\;\bar{Cp}=2.008\;j/g{}^{\circ}C\\ \left[\text{HI}+7.3\;H_{2}O\right],\;\bar{Cp}=1.845\;j/g{}^{\circ}C. \end{array}$$

### 4.5. Heats of Dilution of Sulfuric Acid

The heats of dilution of the sulfuric acid solution by the water included in the aqueous halogen acids have been calculated from existing data [5]. The values obtained correspond to the following processes:

$$\begin{array}{c} 29.1\;H_{2}O\left(\text{liq}\right)+\left[24H_{2}{\text{SO}}_{4}+2600\;H_{2}O+4\text{HI}\right]\left(\text{soln}\right)\\ =\left[24H_{2}{\text{SO}}_{4}+2629.1\;H_{2}O+4\text{HI}\right]\left(\text{soln}\right),\\ \Delta H\left(27{}^{\circ}C\right)=-0.402\pm 0.084\;\text{kj}, \end{array}$$ (5)

$$\begin{array}{c} 20.1\;H_{2}O\left(\text{liq}\right)+\left[24H_{2}{\text{SO}}_{4}+2600\;H_{2}O+4\text{HBr}\right]\left(\text{soln}\right)\\ =\left[24H_{2}{\text{SO}}_{4}+2620.1\;H_{2}O+4\text{HBr}\right]\left(\text{soln}\right)\\ \Delta H\left(25{}^{\circ}C\right)=-0.301\pm 0.084\;\text{kj}, \end{array}$$ (6)

$$\begin{array}{c} 13.5\;H_{2}O\left(\text{liq}\right)+\left[24H_{2}{\text{SO}}_{4}+2600\;H_{2}O+4\text{HCl}\right]\left(\text{soln}\right)\\ =\left[24H_{2}{\text{SO}}_{4}+2613.5\;H_{2}O+4\text{HCl}\right]\left(\text{soln}\right),\\ \Delta H\left(25{}^{\circ}C\right)=-0.230\pm 0.084\;\text{kj}. \end{array}$$ (7)

### 4.6. Heat of Hydrolysis of TiCl_4_

The results of the electrical calibration and TiCl_4_ hydrolysis experiments are given in tables 7 and 8, respectively. The value obtained for the heat of hydrolysis corresponds to the process:

$$\begin{array}{c} {\text{TiCl}}_{4}\left(\text{liq}\right)+\left[24H_{2}{\text{SO}}_{4}+2600\;H_{2}O+4\text{HBr}\right]\left(\text{soln}\right)\\ =\left[{\text{Ti}}^{4+}+4{\text{Cl}}^{-}+4H^{+}+4{\text{Br}}^{-}+24H_{2}{\text{SO}}_{4}+2600\;H_{2}O\right]\left(\text{soln}\right),\\ \Delta H\left(25{}^{\circ}C\right)=-241.81\pm 0.72\;\text{kj}/\text{mole}. \end{array}$$ (8)

### 4.7. Heat of Hydrolysis of TiBr_4_ in Sulfuric Acid Solution Containing Hydrochloric Acid

The results of the electrical calibration and TiBr_4_ hydrolysis experiments are given in tables 9 and 10, respectively. The value obtained for the heat of hydrolysis corresponds to the process:

$$\begin{array}{l} {\text{TiBr}}_{4}\left(c\right)+\left[24H_{2}{\text{SO}}_{4}+2600\;H_{2}O+4\text{HCl}\right]\left(\text{soln}\right)\\ \;=\left[{\text{Ti}}^{4+}+4{\text{Br}}^{-}+4H^{+}+4{\text{Cl}}^{-}+24H_{2}{\text{SO}}_{4}+2600\;H_{2}O\right]\left(\text{soln}\right),\\ \;\Delta H\left(25{}^{\circ}C\right)=-240.80\pm 0.62\;\text{kj}/\text{mole}. \end{array}$$ (9)

### 4.8. Heat of Dilution of Hydrochloric Acid

The results of the hydrochloric acid dilution experiments are given in table 11. As in the case of the hydriodic and hydrobromic acid dilution experiments, the actual calorimetric system was calibrated electrically for each experiment. The quantity of hydrochloric acid was determined by analysis of the resulting solution after each experiment. The value obtained for the heat of dilution corresponds to the process:

$$\begin{array}{r} \left[4\text{HCl}+13.5H_{2}O\right]\left(\text{soln}\right)+\left[24H_{2}{\text{SO}}_{4}+2600H_{2}O\right]\left(\text{soln}\right)\\ =\left[24H_{2}{\text{SO}}_{4}+2613.5H_{2}O+4\text{HCl}\right]\left(\text{soln}\right), \end{array}$$ (10)

ΔH(25°C)=−54.241±0.320kj.

## 5. Heats of Formation

### 5.1. Titanium Tetraiodide Compared to Titanium Tetra bromide

Appropriate combination of eq (1), (2), (3), (4), (5), and (6) leads to the process:

$$\begin{array}{c} {\text{TiI}}_{4}\left(c\right)+\left[4\text{HBr}+20.1\;H_{2}O\right]\left(\text{soln}\right)+9H_{2}O\left(\text{liq}\right)\\ ={\text{TiBr}}_{4}\left(c\right)+\left[4\text{HI}+29.1\;H_{2}O\right]\left(\text{soln}\right),\\ \Delta H\left(27{}^{\circ}C\right)=+6.00\pm 0.54\;\text{kj}. \end{array}$$ (11)

The heat capacities of the aqueous hydrobromic and hydriodic acid solutions, for the range 25° to 27°C, have been taken as 2.008 and 1.845 j/g°C, respectively, from section 4.4 of this paper. The heat capacities of TiBr_4_ and TiI_4_ have been taken as 0.358 and 0.235 j/g°C, respectively. By means of these data we obtain for eq (11):

ΔH(25°C)=+6.29±0.55kj.

The heat of formation of HI in [HI + 7.3 H_2_O](soln) was taken as −50.66 ± 0.04 kj/mole [5], and of HBr in [HBr±5.0 H_2_O](soln) as −109.96±0.18 kj/mole [5].

Thus, we obtain for the difference in heats of formation of TiI_4_ and TiBr_4_:

$$\begin{array}{r} {\text{TiI}}_{4}\left(c\right)+2{\text{Br}}_{2}\left(\text{liq}\right)={\text{TiBr}}_{4}\left(c\right)+2I_{2}\left(c\right),\\ \Delta H\left(25{}^{\circ}C\right)=-230.91\pm 0.75\;\text{kj}/\text{mole}\\ =-55.19\pm 0.18\;\text{kcal}/\text{mole}. \end{array}$$ (12)

Since the heat of formation of TiBr_4_(c) has been previously measured [4] by the direct reaction of titanium with bromine [Δ*Hf*(25°C) = −616.72 ± 4.60 kj/mole], we obtain the following value for the heat of formation of TiI_4_:

$$\begin{array}{r} \text{Ti}\left(\text{solid}\right)+2I_{2}\left(c\right)={\text{TiI}}_{4}\left(c\right)\\ \Delta Hf{}^{\circ}\left(25{}^{\circ}C\right)=-385.81\pm 4.64\;\text{kj}/\text{mole},\\ =-92.21\pm 1.11\;\text{kcal}/\text{mole}. \end{array}$$ (13)

### 5.2. Titanium Tetrachloride Compared to Titanium Tetra bromide

The appropriate combination of eq (6), (7), (8), (9), (10), and (4) leads to the process:

$$\begin{array}{c} {\text{TiBr}}_{4}\left(c\right)+\left[4\text{HCl}+13.5H_{2}O\right]\left(\text{soln}\right)+6.6\;H_{2}O\left(\text{liq}\right)\\ ={\text{TiCl}}_{4}\left(\text{liq}\right)+\left[4\text{HBr}+20.1\;H_{2}O\right]\left(\text{soln}\right),\\ \Delta H\left(25{}^{\circ}C\right)=-21.76\pm 1.04\;\text{kj}/\text{mole}. \end{array}$$ (14)

The heat of formation of HCl in [HCl + 3.4 H_2_O] (soln) is taken as −151.21 ± 0.17 kj/mole [5] and of HBr in [HBr + 5.0 H_2_O](soln) is taken as −109.96 ± 0.13 kj/mole [5]. Thus, we obtain for the difference in heats of formation of TiBr_4_ and TiCl_4_:

$$\begin{array}{r} {\text{TiBr}}_{4}\left(c\right)+2{\text{Cl}}_{2}\left(g\right)={\text{TiCl}}_{4}\left(\text{liq}\right)+2{\text{Br}}_{2}\left(\text{liq}\right),\\ \Delta H\left(25{}^{\circ}C\right)=-186.76\pm 1.34\;\text{kj}/\text{mole},\\ =-44.64\pm 0.32\;\text{kcal}/\text{mole}. \end{array}$$ (15)

This value is in very good agreement with −44.72 ±1.28 kcal for the difference in the directly determined heats of formation of TiBr_4_ (Δ*Hf* = −147.40 ± 1.10) and TiCl_4_ (Δ*Hf* = −192.12 ± 0.65) [3, 4]. This gives added confirmation to the values obtained for the heats of formation of TiCl_4_ and of TiBr_4_.

Gross, Hayman, and Levi [6, 7] have also reported direct determinations of the heats of formation of TiBr_4_ (Δ*Hf* = −148.10 ± 0.25 kcal/mole) and TiCl_4_ (Δ*Hf* = −191.45 ± 0.30 kcal/mole). The difference between these values is −43.35 ± 0.35 kcal/mole, which is in reasonably good agreement with the value obtained in the present investigation.

## Figures and Tables

**Table 1:** Electrical calibration of the *TiI_4_* system

Experiment	*E*	Δ*Rc*	*E_s_*
			
	*j*	*Ohm*	*j*/*ohm*
1	4559.62	0.208827	21834.4
2	4554.26	.208568	21833.3
3	4541.07	.208038	21828.4
4	4591.31	.210309	21831.3
5	4541.72	.208048	21830.3
	
Mean			21831. 5
Standard deviation of the mean			±1.1

**Table 2:** Results of the experiments on the hydrolysis of *TiI_4_*

Experiment	Δ*e*	Δ*RC*	*q*	TiI_4_	−Δ*H*(27° C)
					
	*j*/*ohm*	*Ohm*	*j*	*Mole*	*kj*/*mole*
1	24.9	0.073937	1616.00	0.00742699	217.58
2	27.5	.082475	1802.82	.00826689	218.08
3	29.4	.082103	1794.84	.00822223	218.29
4	26.5	.071992	1573.60	.00724680	217.14
5	29.9	.100405	2194.99	.01009720	217.39
6	25.0	.065496	1431.51	.00655530	218.37
	
Mean					217.81
Standard deviation of the mean					±0.21

**Table 3:** Electrical calibration of *TiBr_4_* system

Experiment	*E*	Δ*Rc*	*E_s_*
			
	*j*	*Ohm*	*j*/*ohm*
1	2064.70	0.094642	21815.9
2	2460.74	.112853	21804.8
3	2458.30	.112683	21816.1
4	2459.08	.112730	21813.9
5	2458.50	.112733	21808.2
	
Mean			21811.8
Standard deviation of the mean			±2.2

**Table 4:** Results of the experiments on the hydrolysis of *TiBr_4_*

Experiment	Δ*e*	Δ*Rc*	*q*	TiBr_4_	−Δ*H*(27° C)
					
	*j*/*ohm*	*Ohm*	*j*	*Mole*	*kj*/*mole*
1	11.9	0.091141	1989.32	0.00812468	244.85
2	14.8	.110283	2407.12	.00984278	244.56
3	14.0	.111086	2424.65	.00992151	244.38
4	13.6	.100716	2198.31	.00898268	244.73
	
Mean					244.63
Standard deviation of the mean					±0.10

**Table 5:** Heat of dilution of aqueous hydriodic acid in 1–N sulfuric acid

Experiment	*E_a_*	Δ*Rc*	*q*	HI	−Δ*H*(27°C)
					
	*j*/*ohm*	*Ohm*	*j*	*Mole*	*kj*/*mole*
1	22100.4	0.001374	30.37	0.0109470	2.774
2	21975.1	.000824	18.11	.0064166	2.822
3	22082.4	.000776	17.14	.0060461	2.835
4	22075.3	.000572	12.63	.0044313	2.850
5	20183.8	.C03105	62.67	.022347	2.804
6	20097.1	.003661	73.58	.026262	2.802
	
Mean					2.814
Standard deviation of the mean					±0.011

**Table 6:** Heat of dilution of aqueous hydrobromic acid in 1–N sulfuric acid

Experiment	*E_a_*	Δ*Rc*	*q*	HBr	−Δ*H*(27°C)
					
	*j*/*ohm*	*Ohm*	*j*	*Mole*	*kj*/*mole*
1	22098.7	0.005812	128.44	0.015962	8.047
2	22073.3	.003218	71.03	.0088925	7.988
3	20150.6	.014119	284.51	.036083	a(7.885)
4	19801.4	.012594	249.38	.031394	7.944
	
Mean					7.993
Standard deviation of the mean					±0.030

a Experiment number 3 was performed at 25° C rather than at 27°C, and the value was not included in calculating the mean. The value at 25° C is required for calculations in sec. 5.2. of this paper. The value of −Δ*H* at 25° C was taken as 7.885 kj/mole (from experiment number 3). This value is in good agreement with that calculated from the mean value at 27° C, using an estimated Δ *Cp* correction.

**Table 7:** Electrical calibration of the *TiCl_4_* system

Experiment	*E*	Δ*Rc*	*E_s_*
			
	*j*	*Ohm*	*j*/*ohm*
1	2907.27	0.133216	21823.4
2	2909.58	.133348	21819.5
3	2903.97	.133034	21828.8
4	2910.32	.133377	21820.3
5	2903.01	.133112	21808.8
6	2900.78	.133003	21809.9
	
Mean			21818.4
Standard deviation of the mean			±3.2

**Table 8:** Results of the experiments on the hydrolysis of *TiCl_4_*

Experiment	Δ*e*	Δ*Rc*	*q*	TiCl_4_	− Δ*H*(27° C)
					
	*j*/*ohm*	*Ohm*	*j*	*Mole*	*kj*/*mole*
1	12.5	0.087154	1902.65	0.0078529	242.29
2	15.7	.113947	2487.93	.0102626	242.43
3	20.7	.146828	3206.59	.0132998	241.10
4	23.0	.162914	3558.27	.0147774	240.79
5	12.8	.089692	1958.08	.0080773	242.42
	
Mean					241.81
Standard deviation of the mean					±0.36

**Table 9:** Electrical calibration of the *TiBr_4_* system

Experiment	*E*	Δ*Rc*	*E_s_*
			
	*j*	*Ohm*	*j*/*ohm*
1	2910.17	0.132796	21914.6
2	2907.41	.132761	21899.6
3	2902.85	.132508	21907.0
4	2902.56	.132474	21910.4
5	2920.41	.133284	21911.2
	
Mean			21908.6
Standard deviation of the mean			±2.5

**Table 10:** Results of the experiments on the hydrolysis of *TiBr_4_*

Experiment	Δ*e*	Δ*Rc*	*q*	TiBr_4_	−Δ*H*(25°C)
					
	*j*/*ohm*	*Ohm*	*j*	*Mole*	*kj*/*mole*
1	13.7	0.122050	2675.62	0.0111647	239.65
2	19.4	.146890	3221.00	.0133545	241.19
3	15.0	.129186	2832.22	.0117395	241.26
4	11.0	.093681	2053.45	.0085122	241.24
5	10.8	.099166	2173.66	.0090327	240.64
	
Mean					240.80
Standard deviation of the mean					±0.31

**Table 11:** Heat of dilution of hydrochloric acid in 1–N sulfuric acid

Experiment	*E_a_*	Δ*Rc*	*q*	HCl	−Δ*H*(25°C)
					
	*j*/*ohm*	*Ohm*	*j*	*Mole*	*kj*/*mole*
1	22129.1	0.025049	554.31	0.041259	13.435
2	22125.3	.024841	549.61	.040435	13.592
3	22129.8	.024867	550.30	.040749	13.505
4	22110.0	.022551	498.60	.036502	13.659
5	22111.9	.023629	522.48	.038246	13.610
	
Mean					13.560
Standard deviation of the mean					±0.040

## Footnotes

1 Figures in brackets indicate the literature references at the end of this paper.
